# Parameters of Vitamin D Metabolism in Patients with Hypoparathyroidism

**DOI:** 10.3390/metabo12121279

**Published:** 2022-12-16

**Authors:** Artem Zhukov, Alexandra Povaliaeva, Zaur Abilov, Elena Kovaleva, Liliya Usoltseva, Anna Eremkina, Vitaliy Ioutsi, Larisa Dzeranova, Ekaterina Pigarova, Liudmila Rozhinskaya, Natalia Mokrysheva

**Affiliations:** Endocrinology Research Centre, 117292 Moscow, Russia

**Keywords:** vitamin D, hypoparathyroidism, vitamin D metabolism, mass-spectrometry, cholecalciferol, alfacalcidol, calcitriol

## Abstract

Only a few studies evaluating the metabolism of vitamin D in patients with hypoparathyroidism (HypoPT) have been performed thus far, and, in particular, they mainly investigated the process of vitamin D activation (specifically, 1α-hydroxylation). This study, therefore, aimed to evaluate the extended spectrum of vitamin D metabolites in patients with HypoPT compared to healthy individuals. We examined 38 adult patients with chronic HypoPT in comparison to 38 healthy adults. The assessment included biochemical parameters (total calcium, albumin, phosphorus, creatinine, and magnesium), parathyroid hormone (PTH), and vitamin D metabolites (25(OH)D_3_, 25(OH)D_2_, 1,25(OH)_2_D_3_, 3-epi-25(OH)D_3_, and 24,25(OH)_2_D_3_) in serum. Our data show that an adequate level of 25(OH)D_3_ (median 35.3 (29.6; 42.0) ng/mL) is achieved with standard doses of cholecalciferol (median 2000 (2000; 2500) IU per day) in HypoPT patients. They also presented with supraphysiological levels of 1,25(OH)_2_D_3_ (median 71 (47; 96) vs. 40 (34; 59) pg/mL, *p* < 0.001) and the increased production of inactive metabolite (median 24,25(OH)_2_D_3_ 3.8 (3.0; 5.1) vs. 1.9 (1.3; 2.7) ng/mL, *p* < 0.001; median 25(OH)D_3_/24,25(OH)_2_D_3_ ratio 8.9 (7.6; 11.1) vs. 13.5 (11.1; 17.0), *p* < 0.001) as compared to the control group. This might be a consequence of the therapy received (treatment with activated vitamin D) and the pathophysiology of the disease (lack of PTH). The abnormality of vitamin D metabolism does not seem to interfere with the achievement of hypoparathyroidism compensation.

## 1. Introduction

Hypoparathyroidism (HypoPT) is a rare condition characterized by reduced parathyroid hormone (PTH) levels resulting in hypocalcemia and hyperphosphatemia. Hypocalcemia leads to increased neuromuscular irritability causing muscle spasms, tingling, and seizures. The most common cause of HypoPT is anterior neck surgery. Less commonly, HypoPT occurs as a result of autoimmune diseases and is secondary to other conditions. To date, HypoPT therapy remains a challenge, and data on optimal treatment strategies for patients with HypoPT are limited due to the lack of controlled clinical trials. Conventional therapy includes activated vitamin D and calcium supplements; however, this treatment does not adequately compensate for the lack of PTH [1]. Inadequate medical control of the disease can lead to both hypocalcemia and hypercalcemia, which might result in long-term complications such as nephrocalcinosis, kidney stones, and calcification of the brain nuclei, vasculature, and other organ systems [1,2]. Recombinant human PTH can also be used as replacement therapy in severe cases [1].

The metabolism of vitamin D occurs in three main steps—25-hydroxylation, 1α-hydroxylation, and 24-hydroxylation. All steps are carried out by cytochrome P450-dependent oxidases [3]. The first step of hydroxylation occurs mainly in the liver via the CYP2R1 enzyme, resulting in the formation of 25(OH)D [4]. In the second step, 1,25(OH)_2_D (calcitriol) is produced by 1α-hydroxylation in the kidneys via the CYP27B1 enzyme (1α-hydroxylase) which is regulated by PTH, fibroblast growth factor 23 (FGF23), and calcitriol itself. PTH stimulates, whereas FGF23 and 1,25(OH)_2_D inhibit CYP27B1 [3,4]. The extrarenal production of calcitriol is possible and has been shown in studies on uremic and anephric patients, where circulating calcitriol levels were determined in the absence of functioning renal tissue [5,6,7]. The expression of CYP27B1 has been detected in keratinocytes, macrophages, and a number of other tissues. It is thought that locally produced 1,25(OH)_2_D acts in autocrine or paracrine manners and is regulated by cytokines [3,4]. 24-hydroxylation via CYP24A1 (24-hydroxylase) leads to the degradation of 1,25(OH)_2_D and 25(OH)D by forming 1,24,25(OH)_3_D and 24,25(OH)_2_D. As such, this step is considered necessary in order to prevent the toxic effects of vitamin D excess. Of particular interest currently are the C3-epimers of vitamin D, which are derived from the conversion of the hydroxyl group in the C3 position of the A-ring from an α to a β orientation [4]. The physiological role of epimers is not fully understood, however, it is reported that 3-epi-1,25(OH)_2_D may possess a calcimimetic effect [8,9].

There are only a few studies that have investigated vitamin D metabolism in HypoPT and were aimed predominantly at the evaluation of the 1α-hydroxylation process. In HypoPT, the 1α-hydroxylation of 25(OH)D in kidneys is impaired due to the lack of the PTH stimulatory effect [10]. However, this process might still take place due to a PTH-independent mechanism of extrarenal hydroxylation [11]. In the study by Lund et al., only a moderate decrease in 1,25(OH)_2_D level as well as its dependence on 25(OH)D concentration was observed in patients with HypoPT [12]. In addition, 25(OH)D has shown pharmacological effects that are typical for the active form of vitamin D in high concentration settings [13].

The aim of this study is thus to evaluate the extended spectrum of vitamin D metabolites in patients with HypoPT compared to healthy individuals.

## 2. Materials and Methods

### 2.1. Study Population and Design

This exploratory study included 38 adult patients with chronic HypoPT admitted for inpatient treatment at a single federal tertiary care center between 2016 and 2022. The diagnosis of HypoPT was established according to the guidelines [14]. The patients were further assigned to compensated (c-HypoPT, n = 13) and non-compensated (nc-HypoPT, n = 25) groups; the compensated state was defined as albumin-adjusted calcium 2.1–2.3 mmol/L and no symptoms of hypocalcemia. The control group included 38 healthy adults. The exclusion criteria were the presence of granulomatous disease, malabsorption syndrome, or liver failure; decreased glomerular filtration rate (less than 60 mL/min per 1.73 m^2^) for both groups; or hypercalcemia, calcium, or vitamin D supplementation for 3 months prior to the study for the control group.

The assessment included biochemical parameters (total calcium, albumin, phosphorus, creatinine, and magnesium), PTH, and vitamin D metabolites (25(OH)D_3_, 25(OH)D_2_, 1,25(OH)_2_D3, 3-epi-25(OH)D_3_, and 24,25(OH)_2_D_3_) in serum. The albumin-adjusted serum calcium levels were calculated using the formula: total plasma calcium (mmol/L) = measured total plasma calcium (mmol/L) + 0.02 × (40 − measured plasma albumin (g/L)) [15].

### 2.2. Sociodemographic and Anthropometric Data Collection

Anthropometric and clinical data were collected from the patient’s medical records.

### 2.3. Laboratory Measurements

The serum samples were stored at −80 °C, avoiding repeated freeze-thaw cycles.

The PTH levels were evaluated by an electrochemiluminescence immunoassay (ELECSYS, Roche, Basel, Switzerland). The biochemical parameters were assessed using an ARCHITECT c8000 analyzer (Abbott, Chicago, IL, USA) and the reagents from the same manufacturer via standard methods.

The levels of the vitamin D metabolites (25(OH)D_3_, 25(OH)D_2_, 1,25(OH)_2_D_3_, 3-epi-25(OH)D_3,_ and 24,25(OH)_2_D_3_) were determined by ultra-high performance liquid chromatography in combination with tandem mass spectrometry (UPLC-MS/MS). The previously in-house developed method [16] has been changed by introducing SelexION Technology in order to avoid the 4-Phenyl-1,2,4-triazoline-3,5-dione (PTAD) derivatization stage leading to spectrometer contamination. A detailed description of the experimental procedure is provided in Appendix A. With this technique, the laboratory participates in DEQAS (the Vitamin D External Quality Assessment Scheme) quality assurance program (lab code 2388), and the results fall within the target range for the analysis of 25(OH)D and 1,25(OH)_2_D metabolites in human serum. All the UPLC-MS/MS measurements were performed after the first successful completion (5/5 samples within the target range) of the DEQAS distributions for both analytes simultaneously. Each batch contained control samples (analytes in blank serum) with both high and low analyte concentrations. The serum samples under investigation were barcoded and randomized prior to the measurements to eliminate analyst-related errors.

### 2.4. Statistical Analysis

The statistical analysis was performed using Jamovi version 2.2.5.0 (The Jamovi Project, 2021). All data were analyzed with non-parametric statistics and are expressed as a median (interquartile range) unless otherwise specified. The Mann–Whitney U-test and Fisher’s exact test were used for comparisons between the two groups. The Spearman rank correlation method was used to obtain coefficients of correlation among indices. A *p*-value of less than 0.05 was considered statistically significant.

## 3. Results

### 3.1. Characteristics of the Groups

The groups were similar in age, sex, and body mass index (BMI) (*p* > 0.05) (Table 1).

The characteristics of the study group in terms of the underlying disease are presented in Table 2. A total of 36 patients were diagnosed with post-surgical HypoPT due to thyroidectomy, 1 patient had HypoPT due to DiGeorge syndrome, and 1 patient had autoimmune polyglandular syndrome type 1. The median duration of the disease in the study group was 61.5 months (27.3; 96.8). In total, 36 (95%) patients received calcium (median dose 2000 (1500; 3000) mg/per day), 33 (87%) patients received alfacalcidol (median dose 1.5 (1.0; 2.0) mcg/per day), 29 patients (76%) received cholecalciferol (median dose 2000 (450; 2500) IU/per day), and 1 patient received teriparatide.

### 3.2. Laboratory Evaluation 

A comparison between HypoPT and control groups is shown in Table 3.

The patients in the HypoPT group had significantly lower PTH, total calcium, albumin-adjusted calcium, and magnesium levels, while their phosphorus levels were higher than in the control group (*p* < 0.05). The rest of the biochemical parameters showed no clinically significant difference. Patients with HypoPT also presented with higher levels of 25(OH)D_3_, 1,25(OH)_2_D_3_, 3-epi-25(OH)D_3_, and 24,25(OH)_2_D_3_ levels, as well as lower 25(OH)D_3_/24,25(OH)_2_D_3_ ratios (*p* < 0.05). Magnesium and creatinine levels showed a positive correlation with albumin-adjusted calcium (r = 0.336, *p* = 0.039 and r = 0.535, *p* < 0.001, respectively). There was also a negative correlation between the disease duration of HypoPT and 24,25(OH)_2_D_3_ and 1,25(OH)_2_D_3_ levels (r = −0.328, *p* = 0.044 and r = −0.424, *p* = 0.008, respectively). The dose of cholecalciferol was positively correlated with 24,25(OH)_2_D_3_ and the calcium dose negatively correlated with 25(OH)D_3_ (r = 0.484, *p* = 0.002 and r = −0.347, *p* = 0.033, respectively).

A comparison between the c-HypoPT group and the nc-HypoPT group is shown in Table 4.

The nc-HypoPT group had significantly lower levels of total calcium, albumin-adjusted calcium, magnesium, and creatinine (*p* < 0.05). There was no significant difference in the levels of PTH, vitamin D metabolites, and the rest of the biochemical parameters.

In the nc-HypoPT group, a negative correlation was found between the duration of HypoPT and magnesium levels (r = −0.461, *p* = 0.021). The significant positive correlation was found between 1,25(OH)_2_D_3_ levels and 25(OH)D_3_, 3-epi-25(OH)D_3_, and 24,25(OH)_2_D_3_ levels (r = 0.694, *p* < 0.001; r = 0.564, *p* = 0.004; and r = 0.416, *p* = 0.040, respectively). The dose of cholecalciferol was positively correlated with 24,25(OH)_2_D_3_ (r = 0.447, *p* = 0.025).

## 4. Discussion

We found that an adequate level of 25(OH)D_3_ was achieved with standard doses of cholecalciferol in HypoPT patients. Almost three-quarters of the included patients had 25(OH)D_3_ levels in the target range (>30 ng/mL), which is slightly higher than the data from the federal registry [21,22] and consistent with some of the previous studies in hypoparathyroidism [23], though some of the studies showed higher levels of 25(OH)D [24]. It is worth noting that the recommended doses of cholecalciferol differ in the guidelines for the general population [17,18] and HypoPT patients [14,25]. The guidelines for HypoPT patients recommend cholecalciferol supplementations in a daily dose of 400–800 IU to the patients treated with activated vitamin D which, given our results, may not be enough to achieve the target range and may need to be revised.

The observed higher concentration of 1,25(OH)_2_D_3_ in the HypoPT group (more than half of the patients had calcitriol levels above the upper limit of the reference range) is presumably related to the intake of activated vitamin D, which is converted to calcitriol by 25-hydroxilase [12]. The levels observed are also higher than the data from other studies in hypoparathyroidism [23,24,26], despite similar dosages of activated vitamin D preparations, which might be influenced by the difference in the methods used for 1,25(OH)_2_D_3_ assessment. This disparity emphasizes the difficulty of the direct comparison of the results obtained by different methods and the importance of using a method for the determination of 1,25(OH)_2_D_3_ that has passed an external quality control system, especially in clinical practice [27]. We have not found a correlation between the 1,25(OH)_2_D_3_ levels and either alfacalcidol dosages or calcium levels, which is consistent with other studies [12,23,26]

The achievement of adequate levels of 25(OH)D_3_ in hypoparathyroidism may be facilitated by decreased 1α-hydroxylation as a result of the lack of PTH. On the other hand, due to the large amounts of active vitamin D, an increase in 24-hydroxylase activity might be expected, leading to the increased degradation of 25(OH)D_3_ in order to prevent toxicity [3,4]. This hypothesis is supported by the higher 24,25(OH)_2_D_3_ levels and lower 25(OH)D_3_/24,25(OH)_2_D_3_ ratios observed in the HypoPT group. Moreover, the higher levels of 25(OH)D_3_ in the HypoPT group might contribute to the obtained differences.

The differences in 3-epi-25(OH)D_3_ between the groups correlated with the observed differences in the 25(OH)D_3_ levels. The biochemical findings were generally expected for this nosology.

We found no difference in the levels of vitamin D metabolites in HypoPT patients depending on compensation status. The observed correlations in nc-HypoPT were consistent with normal vitamin D metabolism and reflected the function of the inactivating metabolic pathways in the presence of high levels of calcitriol. Thus, we assume that the abnormality of vitamin D metabolism does not interfere with the achievement of hypoparathyroidism compensation. We also found that magnesium levels were lower in nc-HypoPT, which highlights the importance of monitoring this parameter according to clinical guidelines [14,25].

Overall, the results do not demonstrate the rationale for determining metabolites in clinical practice in HypoPT patients who fail to achieve compensation from this standpoint. At the same time, other authors conclude that the routine measurement of 1,25(OH)_2_D_3_ may be useful as a biomarker to predict the absence of hypercalciuria in HypoPT patients who are receiving treatment with oral calcium and calcitriol supplements [26].

It should be noted that, to the best of the authors’ knowledge, this is the first cohort study to evaluate the process of vitamin D inactivation in hypoparathyroidism, and the results should be confirmed in further studies with the assessment of a wider range of metabolites in large samples.

Our study had several strengths: the presence of the control group, the comprehensive spectrum of vitamin D metabolism parameters investigated, and participation in an external quality control program for vitamin D metabolite measurements.

However, the study also had some limitations. The small sample size of the groups cautions against the extrapolation of results obtained. In addition, the proportion of women in the groups is significantly higher than in the general population; however, this corresponds to the data from the federal HypoPT registry and can be attributed to the specificity of the disease incidence [21,22]. Some of the important parameters related to mineral metabolism (including FGF23), as well as free 25(OH)D, were not measured. Some additional insights might be obtained given the ability to assess alfacalcidol concentration, which was not possible in the current study. Finally, the proportion between 1,25(OH)_2_D_3_ converted from alfacalcidol and 25(OH)D is unknown.

## 5. Conclusions

This is the first comprehensive study of vitamin D metabolites in patients with hypoparathyroidism. Our data show the supraphysiological levels of 1,25(OH)_2_D_3_ and the increased production of the principal inactive metabolite (24,25(OH)_2_D_3_) in patients with hypoparathyroidism compared to healthy adults which might be a consequence of the received therapy and the pathophysiology of the disease. The abnormality of vitamin D metabolism does not seem to interfere with the achievement of hypoparathyroidism compensation.

## Figures and Tables

**Table 1:** General characteristics of the patients.

	HypoPT			Control (n = 38)	*p* *
	Total (n = 38)	c-HypoPT (n = 13)	nc-HypoPT (n = 25)		
Age, years	49.6 (38.0; 60.0)	53.0 (45.6; 57.9)	46.1 (37.6; 60.8)	48.8 (39.9; 55.5)	*p*_1–4_ = 0.578 *p*_2–3_ = 0.644
Sex (woman, %)	89.5	92.3	88	78.9	*p*_1–4_ = 0.346 *p*_2–3_ = 1.000
BMI, kg/m^2^	27.1 (24.3; 31.6)	28.6 (23.6; 32.2)	27.0 (24.4; 31.4)	25.2 (23.1; 28.4)	*p*_1–4_ = 0.113 *p*_2–3_ = 0.644

* *p*_1–4_—Total and control group comparison; *p*_2–3_—c-HypoPT and nc-HypoPT group comparison. Abbreviations: HypoPT—hypoparathyroidism, c-HypoPT—compensated hypoparathyroidism, nc-HypoPT—non-compensated hypoparathyroidism, BMI—body mass index.

**Table 2:** Characteristics of the patients with hypoparathyroidism in terms of the underlying disease.

	c-HypoPT (n = 13)	nc-HypoPT (n = 25)	*p*
Duration of HypoPT, months	61.0 (35.0; 83.0)	62.0 (19.0; 98.0)	0.794
**Received Therapy** Alfacalcidol, mcg/per day Calcium, mg/per day Cholecalciferol, IU/per day	2.0 (1.0; 2.25) 2000 (1500; 3000) 2000 (2000; 2500)	1.5 (1.0; 2.0) 2000 (1500; 3000) 1200 (0; 2100)	0.306 0.575 0.060

Abbreviations: c-HypoPT—compensated hypoparathyroidism, nc-HypoPT—non-compensated hypoparathyroidism.

**Table 3:** Results of the laboratory evaluation in hypoparathyroidism and control groups.

Laboratory Parameter	Total HypoPT (n = 38)	Control (n = 38)	*p*	Reference Range
Total calcium, mmol/L	2.10 (1.93; 2.25)	2.36 (2.31; 2.43)	* <0.001	2.15–2.55
Albumin-adjusted calcium, mmol/L	2.01 (1.85; 2.15)	2.25 (2.21; 2.31)	* <0.001	2.15–2.55
Phosphorus, mmol/L	1.54 (1.41; 1.76)	1.18 (1.06; 1.23)	* <0.001	0.74–1.52
PTH, pg/mL	5.8 (4.3; 9.3) (n = 29)	38.4 (34.9; 42.1)	* <0.001	15–65
Creatinine, µmol/L	77.2 (72.6; 87.0)	71.5 (65.7; 81.8)	* 0.041	63–110 (male) 50–98 (female)
Albumin, g/L	44.8 (42.3; 46.3)	45.0 (44.1; 46.7)	0.188	35–50
Magnesium, mmol/L	0.75 (0.71; 0.80)	0.81 (0.78; 0.85)	* <0.001	0.7–1.05
25(OH)D_3_, ng/mL	35.3 (29.6; 42.0)	25.1 (17.9; 31.3)	* <0.001	>30 ^1^
3-epi-25(OH)D_3_, ng/mL	3.3 (2.5; 4.5)	2.1 (1.4; 2.6)	* <0.001	Not available
1,25(OH)_2_D_3_, pg/mL	71 (47; 96)	40 (34; 59)	* <0.001	25–66 ^2^
24,25(OH)_2_D_3_, ng/mL	3.8 (3.0; 5.1)	1.9 (1.3; 2.7)	* <0.001	0.5–5.6 ^2^
25(OH)D_3_/24,25(OH)_2_D_3_	8.9 (7.6; 11.1)	13.5 (11.1; 17.0)	* <0.001	7–23 ^2^

^1^ Reference range is given for total 25(OH)D according to the clinical guidelines [17,18]; the 25(OH)D_2_ fraction is negligible (<0.5 ng/mL in absolute values) for the purposes of this study. ^2^ Reference ranges are given according to the literature data [19,20]. * Significant difference in between-group comparison. Abbreviations: HypoPT—hypoparathyroidism, PTH—parathyroid hormone.

**Table 4:** Results of the laboratory evaluation in compensated and non-compensated groups.

Laboratory Parameter	c-HypoPT (n = 13)	nc-HypoPT (n = 25)	*p*	Reference Range
Total calcium, mmol/L	2.26 (2.25; 2.32)	2.01 (1.85; 2.10)	* <0.001	2.15–2.55
Albumin-adjusted calcium, mmol/L	2.18 (2.15; 2.26)	1.93 (1.79; 2.00)	* <0.001	2.15–2.55
Phosphorus, mmol/L	1.51 (1.31; 1.64)	1.58 (1.44; 1.90)	0.113	0.74–1.52
PTH, pg/mL	6.7 (5.6; 8.6) (n = 11)	5.3 (3.9; 9.9) (n = 18)	0.256	15–65
Creatinine, µmol/L	90.2 (81.1; 97.3)	74.2 (69.1; 81.8)	* 0.002	63–110 (male) 50–98 (female)
Albumin, g/L	45.0 (43.0; 46.7)	44.0 (42.2; 45.6)	0.363	35–50
Magnesium, mmol/L	0.79 (0.74; 0.84)	0.74 (0.68; 0.78)	* 0.032	0.7–1.05
25(OH)D_3_, ng/mL	38.7 (29.8; 45.6)	34.8 (28.6; 40.6)	0.518	>30 ^1^
3-epi-25(OH)D_3_, ng/mL	3.3 (3.0; 5.3)	3.1 (2.3; 3.9)	0.103	Not available
1,25(OH)_2_D_3_, pg/mL	95 (59; 96)	60 (45; 92)	0.234	25–66 ^2^
24,25(OH)_2_D_3_, ng/mL	4.4 (3.1; 5.2)	3.7 (2.9; 4.8)	0.329	0.5–5.6 ^2^
25(OH)D_3_/24,25(OH)_2_D_3_	8.7 (6.9; 10.8)	9.6 (8.0; 11.1)	0.523	7–23 ^2^

^1^ Reference range is given for total 25(OH)D according to the clinical guidelines [17,18]; the 25(OH)D_2_ fraction is negligible (<0.5 ng/mL in absolute values) for the purposes of this study. ^2^ Reference ranges are given according to the literature data [19,20]. * Significant difference in between-group comparison. Abbreviations: c-HypoPT—compensated hypoparathyroidism, nc-HypoPT—non-compensated hypoparathyroidism, PTH—parathyroid hormone.

## Data Availability

The datasets generated during and/or analyzed during the current study are available from the corresponding author upon reasonable request. The data are not publicly available due to due to privacy reasons.

## Appendix A

The levels of vitamin D metabolites in serum were determined by UPLC-MS/MS. The previously in-house developed method [16] has been changed by introducing SelexION Technology in order to avoid the PTAD derivatization stage, leading to spectrometer contamination.

Briefly, a 50 µL mixture of deuterated internal standards (25(OH)D_3_-d_6_, 1,25(OH)_2_D_3_-d_6_, 3-epi-25(OH)D_3_-d_3_, and 24,25(OH)_2_D_3_-d_6_) was added to 300 µL of the serum, vortexed, and equilibrated for 5 min. Then, 900 µL of EtOAc was added for 15 min followed by extraction and centrifugation (14,800 rpm, 6 min, 25 °C); the organic layer was separated and dried (40 °C, 1350 rpm, 5 mbar vacuum). The residues were reconstituted in a 4:6 methanol/water mixture (1 mL) and the resulting liquid was loaded onto Agilent Bond Elut C18 (50 mg, 1 mL) cartridges preconditioned with 0.5 mL methanol and 1 mL water. The cartridges were then washed with water followed by a 3:7 methanol/water mixture (1 mL of each), and analytes were eluted with 2 × 600 µL of methanol. The eluate was evaporated to dryness using a vacuum centrifuge. A total of 115 µL of 1:1 methanol/water mixture was added to residues; after 10 min stirring in a shaker, the samples were centrifugated (14,800 rpm, 10 min, 5 °C) and transferred to the 96-well plate. In total, 80 µL of the samples were injected into the Agilent 1290 Infinity II LC equipped with a 4-channel Flexible pump and Acquity UPLC HSS PFP column (2.1 × 50 mm, particle size 1.8 µm, Waters). The flow rate and column temperature were 0.4 mL/min and 40 °C and the gradient program is present in Table A1.

**Table A1:** Gradient separation program.

Time, min	Acetonitrile, %	Methanol, %	0.1% Formic Acid in Water, %
0	0	50	50
1.0	0	50	50
1.5	0	60	40
2.0	0	60	40
3.2	0	70	30
4.4	3	68	29
4.8	5	70	25
5.0	5	70	25
6.0	5	75	20
6.5	0	85	15
8.0	0	85	15
8.5	0	50	50
12	0	50	50

Detection was provided by AB Sciex QTrap 5500 mass-spectrometer using an ESI source and a SelexION differential mobility separation device. Detection was performed in the positive ion mode, the capillary voltage was set at 5500 V, and the ion source gas pressure, turbo gas, and curtain gas pressure were 50, 60, and 28 psi, respectively; the turbo gas temperature was 650 °C. The parameters of the differential ion mobility system were constant for all components: the separation, compensation, offset voltages, and interface temperature were 3800 V, 6.8 V, −20 V, and 150 °C, and the carrier gas was nitrogen. A carrier gas modifier and the resolution enhancement mode were not used. The registration of the components was carried out in the scheduled multiple reaction monitoring. The time interval for each component was 70 s, the target scan time was 0.7 s, and the entrance potential was kept at 10 V. The other parameters were selected for each component individually and are presented in Table A2. Data were controlled and collected using the Analyst 1.6.3 software (AB Sciex).

**Table A2:** Multiple reaction monitoring transitions used for the detection of vitamin D metabolites.

Analyte	Transition Type	Q1	Q3	tR, min	CE, V	DP, V	CXP, V
1,25(OH)_2_D_3_	quantifier	399.3	135.1	5.0	28	89	16
	qualifier	399.3	381.3		19	89	14
24,25(OH)_2_D_3_	quantifier	417.3	399.3	4.8	13	66	15
	qualifier	417.3	381.3		15	66	14
25(OH)D_3_	quantifier	401.3	383.3	6.1	13	59	15
	qualifier	401.3	365.4		17	59	13
3-epi-25(OH)D_3_	quantifier	401.29	383.2	6.3	14	110	9
	qualifier	401.29	365.3		17	110	9
25(OH)D_2_	quantifier	413.3	355.3	6.3	15	110	7
	qualifier	413.3	395.3		13	110	7
1,25(OH)_2_D_3_-d_6_	IS	405.3	135.0	5	30	170	12
24,25(OH)_2_D_3_-d_6_	IS	423.3	387.5	4.8	16	150	7
25(OH)D_3_-d_6_	IS	407.4	389.3	6.1	12	120	11
3-epi-25(OH)D_3_-d_3_	IS	404.4	368.3	6.3	18	150	6

Abbreviations: Q1—quadrupole 1 mass, Q3—quadrupole 3 mass, tR—retention time, DP—declustering potential, CE—collision energy, CXP—collision energy exit potential.
